# Supplementary material for: Using computational approaches to enhance the interpretation of missense variants in the PAX6 gene
Source: Eur J Hum Genet. 2024 Jun 7;32(8):1005–13. doi: 10.1038/s41431-024-01638-3 (PMC11292026; doi:10.1038/s41431-024-01638-3)
Supplement: Supplementary file 3 — Supplementary Table 3 [file 41431_2024_1638_MOESM3_ESM.pdf]

Supplementary Table 3. Performance of the computational tool combinations assessed in this study (in tasks involving *PAX6* missense variant evaluation).

Combination	Sp (%)	Sn (%)	Acc (%)	PPV (%)	MCC
AlphaMissense + REVEL	96	85	88	98	0.76
AlphaMissense + SIFT4G	96	84	88	98	0.76
REVEL + SIFT4G	97	86	90	99	0.79
AlphaMissense + REVEL + SIFT4G	96	87	90	98	0.78

A genetic variant was considered pathogenic if the scores of ≥ 2 tools surpassed the corresponding optimized thresholds. Sp, specificity; Sn, sensitivity; Acc, Accuracy; PPV, positive predictive value; MCC, Matthews correlation coefficient. All percentages were rounded to zero decimal points.
