# Supplementary material for: Using computational approaches to enhance the interpretation of missense variants in the PAX6 gene
Source: Eur J Hum Genet. 2024 Jun 7;32(8):1005–13. doi: 10.1038/s41431-024-01638-3 (PMC11292026; doi:10.1038/s41431-024-01638-3)
Supplement: Supplementary file 4 — Supplementary Table 4 [file 41431_2024_1638_MOESM4_ESM.pdf]

Supplementary Table 4. Performance of computational tools in the context of a modeled "contaminated" pool of presumed neutral/benign variants (including 17 presumed pathogenic variants from ClinVar and PubMed).

Tool	Gene-specific threshold	Sp (%)	Sn (%)	Acc (%)	PPV (%)	MCC
AlphaMissense	>0.9667	79	88	85	87	**0.67**
BayesDel	> 0.38	73	88	82	84	0.61
CADD	> 25.25	74	85	81	84	0.59
ClinPred	> 0.90	63	93	82	80	0.60
Eigen	> 0.34	49	91	76	75	0.46
MutPred2	> 0.61	66	95	84	82	**0.65**
PolyPhen2	> 0.90	73	81	78	83	0.53
REVEL	> 0.77	74	89	83	85	0.64
SIFT4G	≤ 0.03	77	89	84	86	**0.66**
VEST4	> 0.85	75	73	73	83	0.46

Bold text and blue highlighted cells show the best-performing tools after incorporating presumed pathogenic variants from ClinVar (n=12) and PubMed (n=5) into Primary Dataset Neutral. Sp, specificity; Sn, sensitivity; Acc, accuracy; PPV, positive predictive value; MCC, Matthews correlation coefficient. All percentages were rounded to zero decimal points.
